# Clinical outcomes of teicoplanin use in the OPAT setting

**DOI:** 10.1016/j.ijantimicag.2020.105888

**Published:** 2020-03

**Authors:** Hannah Dabrowski, Helena Wickham, Surjo De, Jonathan Underwood, Stephen Morris-Jones, Sarah Logan, Michael Marks, Gabriele Pollara

**Affiliations:** a Division of Infection, University College London Hospitals, London, UK; b Department of Clinical Microbiology, University College London Hospitals, London, UK; c Department of Infectious Diseases, Cardiff and Vale University Health Board, Cardiff, UK; d Clinical Research Department, London School of Hygiene and Tropical Medicine, London, UK; e Division of Infection & Immunity, University College London, London, UK

**Keywords:** Teicoplanin, Ceftriaxone, OPAT, Infectious diseases

## Abstract

Teicoplanin possesses several convenient properties for use in the delivery of outpatient parenteral antimicrobial therapy (OPAT) services. However, its use is not widespread and data on its efficacy in the OPAT setting are limited. Here we present a case series of patients undergoing OPAT care being treated by either teicoplanin-based (*n* = 107) or ceftriaxone-based (*n* = 191) antibiotic regimens. Clinical failure with teicoplanin occurred in five episodes of care (4.7%) compared with only two episodes of ceftriaxone-based OPAT care (1.0%). Teicoplanin-associated clinical failure was observed in 2 (33.3%) of 6 patients with *Enterococcus* infections compared with 3 (3.0%) of 101 patients with non-*Enterococcus* infections. Overall, there were four (2.9%) drug-related adverse events for teicoplanin and four (1.8%) for ceftriaxone, prompting a switch to teicoplanin in three patients. These findings support the continued use of teicoplanin in OPAT as well as its consideration in centres where it is not currently being offered.

## 1 Introduction

Outpatient parenteral antimicrobial therapy (OPAT) services offer several advantages over conventional inpatient care, including reduced length of inpatient stay, improved patient satisfaction and cost reductions [1,2]. One of the challenges posed by OPAT is the choice of antimicrobial, needing to balance the convenience of a drug's dosing interval with an appropriate safety and efficacy profile [3]. Two commonly used antimicrobial agents in OPAT are ceftriaxone and teicoplanin, with overlapping spectra of activity for Gram-positive infections, although ceftriaxone possesses no activity against *Enterococcus*infections as a single agent [4]. The pharmacokinetics of both agents make them an attractive choice for OPAT as they can be administered once daily, and also three times weekly for teicoplanin [5] Teicoplanin displays equivalent efficacy with reduced nephrotoxicity compared with the other commonly used glycopeptide, vancomycin [6]. Although licensed in Europe and also being widely used in Asia and South America, teicoplanin is not currently approved for use in USA. Yet in the OPAT setting it is effective in managing skin and soft-tissue infections (SSTIs) [7], shows good outcomes for bone and joint infections (BJIs) [8] and it also has a role in the treatment of select cases of endocarditis [9]. However, some groups have reported use of OPAT teicoplanin as an independent risk factor for clinical failure in the management of infective endocarditis [10] and in SSTIs, where 25% of patients experienced treatment failure, and for which ceftriaxone showed superior clinical outcomes [3].

With the continued widespread use of teicoplanin in OPAT [4,[11], [12], [13], [14], such findings compel further review of clinical outcomes with its use across a range of clinical conditions, particularly in comparison with ceftriaxone. Our centre has extensive experience of using teicoplanin [15] and a recent review of our service has revealed it to be the glycopeptide of choice and the second most prescribed OPAT antibiotic after ceftriaxone [13]. In the current study, prospectively collected data were reviewed to assess the clinical outcomes of teicoplanin-based regimens and, where appropriate, to compare with ceftriaxone-based OPAT care.

## 2 Methods

### 2.1 Ethics and data extraction

Patient data, including demographics, antimicrobial(s) administered, drug-associated adverse events (AEs) and microbiological investigations, were extracted from the OPAT electronic Clinical Infectious Diseases (elCID) database at University College London Hospitals (UCLH) NHS Foundation Trust [13]. Clinical records were anonymised at the time of data extraction. The study was approved by the Audit and Research Committee at the Hospital for Tropical Diseases, UCLH, who stated that as this was a retrospective review of routine clinical data being analysed for service development purposes, further formal ethical approval was not required.

### 2.2 Patient cohort selection

The case records of all patients treated by the OPAT team between January 2015 and February 2018 were identified. Only patients who received ≥3 days of teicoplanin or ceftriaxone via the OPAT service were considered. A total of 152 episodes of OPAT care that involved ≥3 days of teicoplanin administration were identified, but the analyses focused on the most common OPAT indications, namely osteomyelitis, SSTI, bacteraemia, endovascular infection and discitis, which accounted for 136 (89.5%) teicoplanin-based episodes (Supplementary Table S1) to allow comparison with ceftriaxone. A total of 31 episodes of care during which both teicoplanin and ceftriaxone were administered, even if not contemporaneously, were identified. Administration of any other antibiotics during the OPAT episode was not an exclusion criterion to enrolment in the study. Unfortunately, patient weight was not routinely recorded, negating display of teicoplanin dosing per kilogram. However, routine practice at our centre is to use 10–12 mg/kg for endovascular infections and BJIs. In addition, therapeutic drug monitoring (TDM) is also performed for teicoplanin weekly, and the final doses of teicoplanin, corrected according to TDM if required, are described in Table 1.

**Table 1:** Teicoplanin dosing for outpatient parenteral antimicrobial therapy (OPAT) care episodes.

Teicoplanin dose (mg)	Frequency	No. (%) of episodes
801–1000	Daily	40 (37.4)
601–800	Daily	22 (20.6)
401–600	Daily	17 (15.9)
400	Daily	10 (9.3)
800–1500	×3 weekly	15 (14.0)
Not documented	–	3 (2.8)

### 2.3 Clinical definitions

All patients were reviewed clinically at least weekly during an OPAT care episode by a multidisciplinary team including at least two infection specialists. The choice of antimicrobials was made based on clinician preference, considering multiple factors including microbial susceptibility data, site of infection, co-morbidities and drug allergy status. Outcomes were determined at the end of the period of intravenous (i.v.) therapy using the standardised National Outcomes Registry System (NORS) definitions (http://opatregistry.com/). Clinically significant drug-related AEs were defined as hospital re-admissions, change of OPAT antimicrobial drug owing to toxicity, or *Clostridioides difficile* infection. Minor changes in biochemical parameters or other minor AEs that did not necessitate admission or change in therapy were excluded. Re-admission was determined as an admission to hospital during the period of OPAT.

### 2.4 Statistical analysis

Continuous variables are described with the median and interquartile range (IQR). Categorical data are described as the number and percentage. The underlying diagnosis was categorised as SSTI, bacteraemia, BJI or endovascular infection (including endocarditis). Microbiology data were categorised regarding the presence or absence of *Staphylococcus aureus*, streptococci, enterococci, coagulase-negative staphylococci and Gram-negative organisms. Given the rarity of the outcome of clinical failure, multivariable analysis was not performed owing to the potential for sparse data.

## 3 Results

### 3.1 Cohort description and exclusions

From the clinical records in the elCID database, 107 episodes where the patient received teicoplanin but no ceftriaxone and 191 episodes where the patient received ceftriaxone but no teicoplanin were found, which were defined as teicoplanin only-based and ceftriaxone only-based OPAT episodes, respectively (Table 2). Patients received both teicoplanin and ceftriaxone of any duration during the clinical episode in 31 episodes (Table 2); there were 3 failures (9.7%) in these patients. One patient on teicoplanin was admitted for a joint washout despite therapeutic levels of teicoplanin but then achieved cure following a further 6 weeks of teicoplanin monotherapy. In another patient the underlying diagnosis of osteomyelitis and causative organism were never confirmed in the context of advanced malignancy. One other patient receiving teicoplanin was admitted with Gram-negative sepsis (Table 3). In 4 of the 31 patients who received both teicoplanin and ceftriaxone a rash developed whilst on ceftriaxone, prompting a switch to teicoplanin in 3 cases. In contrast, 1 patient developed a rash whilst on teicoplanin. Due to the potential confounder of receiving both of these antibiotics during the episode of OPAT care, these 31 were excluded from further analyses of clinical outcome.

**Table 2:** Indications and outcomes of teicoplanin or ceftriaxone use in outpatient parenteral antimicrobial therapy (OPAT)^a^.

	Teicoplanin only (*n* = 107)	Ceftriaxone only (*n* = 191)	Teicoplanin + ceftriaxone (*n* = 31)
Demographics			
Age (years) [median (IQR)]	61.5 (49–79)	63 (45–75)	67 (57.5–75.5)
Sex (M:F)	1.7:1	2.4:1	1.8:1
Diabetes mellitus	15 (14.0)	21 (11.0)	4 (12.9)
HIV-seropositive	2 (1.9)	7 (3.7)	1 (3.2)
Diagnoses			
Osteomyelitis	62 (57.9)	48 (25.1)	17 (54.8)
Bacteraemia	18 (16.8)	0 (0)	3 (9.7)
Endovascular infection (including endocarditis)	12 (11.2)	26 (13.6)	3 (9.7)
SSTI	10 (9.3)	100 (52.4)	5 (16.1)
Discitis/vertebral osteomyelitis	5 (4.7)	17 (8.9)	3 (9.7)
Organisms identified^b^			
CoNS	27 (25.2)	9 (4.7)	2 (6.5)
MSSA	23 (21.5)	58 (30.4)	11 (35.5)
MRSA	8 (7.5)	0 (0)	1 (3.2)
*Streptococcus* spp.	10 (9.3)	20 (10.5)	7 (22.6)
Gram-negative organisms	6 (5.6)	9 (4.7)	1 (3.2)
*Enterococcus* sp.	6 (5.6)	0 (0)	1 (3.2)
Other organisms	2 (1.9)	11 (5.8)	1 (3.2)
No organism identified	32 (29.9)	97 (50.8)	9 (29.0)
Duration of teicoplanin use (days)			
0–2	–	N/A	3 (9.7)
3–7	17 (15.9)		11 (35.5)
8–14	28 (26.2)		6 (19.4)
15–21	12 (11.2)		3 (9.7)
22–28	12 (11.2)		2 (6.5)
≥29	38 (35.5)		6 (19.4)
Duration of ceftriaxone use (days)			
0–2	N/A	–	4 (12.9)
3–7		90 (47.1)	11 (35.5)
8–14		44 (23.0)	5 (16.1)
15–21		22 (11.5)	2 (6.5)
22–28		13 (6.8)	1 (3.2)
≥29		22 (11.5)	8 (25.8)
Teicoplanin-associated adverse events			
Rash	1 (0.9)	N/A	1 (3.2)
Renal impairment	2 (1.9)	N/A	0 (0.0)
Ceftriaxone-associated adverse events			
Rash	N/A	0 (0.0)	4 (12.9)^c^
Patient outcome			
Cure and improved	101 (94.4)	188 (98.4)	27 (87.1)
Failure	5 (4.7)	2 (1.0)	3 (9.7)
Not documented	1 (0.9)	1 (0.5)	1 (3.2)

CoNS, coagulase-negative staphylococci; HIV, human immunodeficiency virus; IQR, interquartile range; MRSA, methicillin-resistant *Staphylococcus aureus*; MSSA, methicillin-susceptible *S. aureus*; N/A, not applicable; SSTI, skin and soft-tissue infection.

a Data are *n* (%) unless otherwise stated.

b Organisms listed were identified by routine microbiological analysis. Antimicrobial drugs used included empirical choices for presumptive involvement of non-identified organisms.

c In three of these patients, the rash prompted a switch to teicoplanin.

**Table 3:** Characterisation of patients who failed outpatient parenteral antimicrobial therapy (OPAT) therapy^a^.

Category	Underlyingdiagnosis	Otherco-morbidity	Age (years)	Sex	DM	HIV	TEC dose	TEC TDM (mg/L)	Organism	Cause of failure	Consequenceof failure
Teicoplanin only	Endovascular infection	Neutropenia secondary to MDS	76	F	No	No	400 mg daily	N/P	*Enterococcus* sp.	Not specified	Died
Teicoplanin only	Endovascular infection (endocarditis)	TAVI, RA, COPD	68	F	No	No	800 mg daily	21	*Enterococcus* sp.	Medical failure for prosthetic valve endocarditis	Aortic valve replacement
Teicoplanin only	Osteomyelitis (prosthetic knee infection)	–	73	M	Yes	No	800 mg daily	23	CoNS	PICC line thrombus	PICC line removal and reassessment
Teicoplanin only	Osteomyelitis (prosthetic shoulder infection)	JRA	28	F	No	No	800 mg daily	42	CoNS and *Candida* sp.	Thrombophlebitis and deranged liver function	Re-admission
Teicoplanin only	Osteomyelitis (left knee replacement)	TAVI	82	M	No	No	1000 mg daily	28	*Streptococcus bovis*	Midline thrombus and prosthetic valve vegetation	Re-admission
Teicoplanin and ceftriaxone	Discitis/vertebral osteomyelitis	–	80	M	No	No	1000 mg daily	34	CoNS	Gram-negative sepsis	Re-admission
Teicoplanin and ceftriaxone	Osteomyelitis (left elbow prosthetic infection)	–	63	M	No	No	800 mg daily	33	*Staphylococcus aureus*	Recurrence of elbow infection	Re-admission
Teicoplanin and ceftriaxone	Osteomyelitis (pelvis)	SCC of the penis	54	M	No	No	1000 mg daily	31	No organism identified	Lack of clinical response with advanced malignancy	Died
Ceftriaxone only	Osteomyelitis (diabetic foot)	–	72	M	Yes	No	–	N/A	*Staphylococcus aureus*	Unable to tolerate i.v. catheter	Re-admission
Ceftriaxone only	Endovascular infection (endocarditis)	TAVI, AF	79	F	No	No	–	N/A	*Abiotrophia defectiva*	Right MCA infarct	Re-admission

AF, atrial fibrillation; CoNS, coagulase-negative staphylococci; COPD, chronic obstructive pulmonary disease; DM, diabetes mellitus; HIV, human immunodeficiency virus; i.v., intravenous; JRA, juvenile rheumatoid arthritis; MCA, middle cerebral artery; MDS, myelodysplasia; N/A, not applicable; N/P, not performed; PICC, peripherally-inserted central catheter; RA, rheumatoid arthritis; SCC, squamous cell carcinoma; TAVI, transcatheter aortic valve implantation.

a Teicoplanin (TEC) dosage and therapeutic drug monitoring (TDM) indicated in the table. All patients on ceftriaxone received a dose of 2 g daily.

### 3.2 Outcomes of teicoplanin-based OPAT care

The median age of patients receiving teicoplanin only-based OPAT care was 61.5 years (IQR 49–79 years) and the male to female ratio was 1.7:1. Of the 107 patients, 15 (14.0%) also had a diagnosis of diabetes mellitus and 2 (1.9%) were human immunodeficiency virus (HIV)-seropositive (Table 2). The median duration of teicoplanin administration was 20 days (IQR 11–35 days) and the mode teicoplanin dose ranged between 800 mg and 1000 mg daily (Tables 1 and 2). Gram-positive bacteria were the most common causative organisms, although teicoplanin was also used in clinical episodes where Gram-negative infections were involved but a polymicrobial infection was suspected (Table 2). Clinically significant teicoplanin-associated AEs were rare, occurring in only three episodes (2.8%) (Table 2). Overall, 101 patients (94.4%) met the NORS definition of cure or clinical improvement and 5 patients (4.7%) met the definition of clinical failure (Table 2). Of the clinical failures, four of the five had achieved therapeutic levels of teicoplanin. Three patients were re-admitted predominantly due to i.v. catheter complications, one required aortic valve replacement following prosthetic valve endocarditis, and one patient died of underlying endovascular infection in the context of neutropenia (Table 3).

### 3.3 Outcomes of ceftriaxone-based OPAT care

The median age of patients receiving ceftriaxone only-based OPAT care was 63 years (IQR 45–75 years) and the male to female ratio was 2.4:1. The most common indication for ceftriaxone-based OPAT care was SSTI, making up just over one-half (100/191; 52.4%) of the episodes (Table 2). No clinically significant AEs were observed in the ceftriaxone only-based cohort, although in 4 patients who also received teicoplanin during their OPAT care episode a rash developed whilst on ceftriaxone. The clinical failure rate was low, with two patients (1.0%) requiring re-admission (one for i.v. catheter-associated complications and the other due to a stroke) (Table 3). Overall, 188 patients (98.4%) were cured or experienced significant improvement following the ceftriaxone-only based OPAT episode of care (Table 2).

### 3.4 Comparison between teicoplanin- and ceftriaxone-based OPAT care

There was no significant difference in the age distribution (*P* = 0.397, Mann–Whitney test) or prevalence of diabetes mellitus (*P* = 0.44, χ^2^ test) between the two populations. However, the median (IQR) duration of ceftriaxone administration [7 (5–15) days] was shorter than for teicoplanin (*P* < 0.0001, Mann–Whitney test), and ceftriaxone was used more frequently than teicoplanin for SSTIs (*P* < 0.0001, χ^2^ test), a diagnosis for which there were no clinical failures throughout the cohort. In addition, ceftriaxone was used more frequently where no causative organism was identified (*P* = 0.018, χ^2^ test) as a means to ensure reasonable bacteriological coverage, but was never used for enterococcal infections (Table 2). These differences preclude direct comparison of the clinical failure rates between teicoplanin- and ceftriaxone-based OPAT care, although this was low in both groups (4.7% and 1.0%, respectively). It was also notable that in the teicoplanin-only group, 2 (33.3%) of 6 patients with an *Enterococcus* infection failed OPAT therapy in contrast to only 3 (3.0%) of 101 with a non-*Enterococcus* infection (Table 3).

## 4 Discussion

Teicoplanin is an attractive antimicrobial to use in the OPAT setting in view of its favourable dosing regimens and safety profile [5,6]. Despite this, its use is not widespread, in part due to the absence of supportive clinical outcome data. Here we report a prospectively recorded cohort detailing our real-world experience of teicoplanin use in an OPAT setting. The most striking finding was the relatively low failure rate compared with that reported previously [3,^10^,16]. Focusing on the same clinical conditions for which teicoplanin was indicated, comparable clinical outcomes with ceftriaxone, another commonly used OPAT antimicrobial, were observed. These data suggest that teicoplanin is a viable option for OPAT.

This study has several strengths, including the broad nature of the cohort and the prospective standardised data collection. A standardised UK NORS definition of clinical outcome in OPAT care was utilised, but this is limited to an assessment of outcome at the end of i.v. antimicrobial therapy. We were unable to obtain outcome data beyond this point, whereas previous studies assessed outcomes 1 month after the end of OPAT care [3,16]. Nevertheless, in these other studies, drug AEs were common (9% of teicoplanin-based episodes) and thus made up a sizeable proportion of causes for OPAT failure [3], an aspect not seen in the current cohort. In contrast, over one-third of clinical failures in the current cohort were related to complications with i.v. catheters, in line with our previous observations that this is a significant source of OPAT AEs [13]. The main analysis focused on patients who had received either teicoplanin only- or ceftriaxone only-based care, but amongst patients who received both drugs significant AEs were seen in four patients receiving ceftriaxone, prompting a switch to teicoplanin in three, whereas there was only one teicoplanin-related AE in this group. The low drug AE rate with teicoplanin is unlikely to be related to dosing, as the dosing used in this cohort was comparable with other studies [3,10] where attained therapeutic levels did not significantly impact upon the rate of AEs [17]. Indeed, we routinely performed TDM for teicoplanin and subtherapeutic levels were not a feature of any of the cases of clinical failure. Many in this cohort of patients had already started i.v. antimicrobials prior to admission onto the OPAT service, and thus early antibiotic-related AEs, occurring during inpatient care, may not have been documented by the OPAT service.

A noticeable feature of this cohort was that osteomyelitis was the most common disease category treated with teicoplanin. This standardised category has been associated with worse OPAT outcomes [18] and includes joint infections both with and without prosthetic material. Despite this heterogeneity, the high success rate of OPAT care both with teicoplanin and ceftriaxone was notable and further supports the treatment of these conditions by OPAT services, especially in the absence of suitable oral regimens [14,19]. Methicillin-resistant *S. aureus* (MRSA) infection has previously been associated with OPAT failures [3], but MRSA was rare in the current study. On the other hand, the current data suggest a relationship between *Enterococcus* infection and OPAT failure, an observation not previously reported. Of interest, a recent retrospective case series found *Enterococcus* infection to be associated with treatment failure for late acute prosthetic joint infections [20]. Evaluation of a larger cohort of patients via the national OPAT registry may allow a more detailed assessment of the association between specific organisms and clinical outcomes.

This study has several limitations. Foremost, as mentioned above, data were collected on clinical outcomes only at the end of OPAT care as part of a standardised national reporting system and not longer-term than this. As relapse of the underlying infection can occur several weeks after ending OPAT care [16], the true rate of clinical failure may have been underestimated. The majority of patients received additional agents before, after or alongside teicoplanin and ceftriaxone, and while this is reflective of real-world practice, it may have limited the ability to detect differences directly attributable to the drugs of interest, particularly when comparing outcomes in patients receiving both teicoplanin and ceftriaxone during the same clinical episode. Moreover, despite no evidence supporting the use of teicoplanin in the elderly or in patients with diabetes, the heterogeneity in clinical conditions and underlying microbiological causes between the cohorts as well as the low rate of clinical failure limited the ability to conduct comparative multivariable statistical analyses of clinical outcomes.

## 5 Conclusions

Here we report a low clinical failure rate from a real-world cohort of patients receiving OPAT care with teicoplanin, a safe and convenient antimicrobial. The data support the continued use of teicoplanin in OPAT and its consideration in centres where it is not currently being offered.

## Appendix Supplementary materials

Image, application 1
